# Effects of platelet-rich plasma injection on electrical activity and biomechanics of the erector spinae muscles in lumbar myofascial pain syndrome

**DOI:** 10.1038/s41598-024-72554-1

**Published:** 2024-09-18

**Authors:** Na Li, Qian Wang, Shaolong Ai, Hongchen He, Jiayuan He, Ning Jiang

**Affiliations:** 1 The National Clinical Research Center for Geriatrics, West China Hospital of Sichuan University, Chengdu, Sichuan Province China; 2 Medical Equipment Innovation Research Center, West China Hospital of Sichuan University, Chengdu, Sichuan Province China; 3 The Med-X Center for Manufacturing, Sichuan University, Chengdu, Sichuan Province China; 4 The Department of Rehabilitation Medicine Center, West China Hospital of Sichuan University, Chengdu, Sichuan Province China

**Keywords:** Myofascial pain syndrome, Surface electromyography, Biomechanical parameters, Platelet-rich plasma, Rehabilitation, Electrodiagnosis

## Abstract

Low back pain (LBP) is a highly prevalent disease. Among the various causes of LBP, one of the most frequent is myofascial pain syndrome (MPS) which affects the spinal stabilizer muscles. The aims of this study were to compare the differences in muscular electrical activity and biomechanical properties between the painful and non-painful sides in patients with unilateral MPS and to verify the feasibility of surface electromyography (sEMG) and MyotonPRO for assisting in MPS assessment. Forty patients with unilateral lumbar MPS were recruited via the Department of Rehabilitation Medicine Center of West China Hospital Sichuan University from October 2022 to October 2023. The electrical properties of the bilateral erector spinae muscles were characterized by sEMG signals during a trunk extension task. The following four time-domain features of sEMG were extracted: root mean square (RMS), mean absolute value (MAV), integrated EMG (iEMG), and waveform length (WL). And two frequency domain features were extracted: the median frequency (MDF) and mean power frequency (MPF). The mechanical properties of the muscles were assessed by MyotonPRO at rest. The following biomechanical parameters were acquired: oscillation frequency [Hz], dynamic stiffness [N/m], logarithmic decrement, relaxation time [ms], and Creep. The visual analog scale (VAS) was used to evaluate the pain severity, and the Oswestry Disability Index (ODI) was used to evaluate the severity of disability and disruption to lifestyle activities caused by LBP pain. The outcome measures were obtained prior to the Platelet-rich plasma (PRP) treatment and repeated two weeks after treatment. (1) Prior to the PRP treatment, all sEMG time-domain features on the painful side were significantly higher than those on the non-painful side (RMS, *p* < 0.001; MAV, *p* < 0.001; iEMG, *p* < 0.001; WL, *p* = 0.001). However, there was no significant difference in the sEMG frequency-domain features (MPF, *p* = 0.478; MDF, *p* = 0.758). On the mechanical side, there were significant differences in oscillation frequency (*p* = 0.041) and logarithmic decrement (*p* = 0.022) between the painful side and non-painful side, but no significant differences in dynamic stiffness, relaxation time, and creep (both *p* > 0.05). (2) Two weeks after the PRP treatment, statistically significant decreases were observed in both post-treatment VAS (*p* < 0.001) and ODI scales (*p* < 0.001), indicating the PRP treatment clinically significantly reduced the level of. MPS. This change coincided with all sEMG time-domain features, in which the values at the painful side decreased significantly (RMS, *p* = 0.001; MAV, *p* = 0.001; iEMG, *p* = 0.001; WL, *p* = 0.001). However, no significant difference in the sEMG frequency-domain features (MPF, *p* = 0.620; MDF, *p* = 0.850) was found. On the mechanical side, only logarithmic decrement on the painful side increased significantly (*p* < 0.001). Our combined MyotonPRO and sEMG results indicated that MPS likely leads to increased muscle tone and decreased muscle elasticity, manifested by abnormal time-domain features of sEMG and biomechanical properties. The changes in these objective measurements were agreed with the changes in subjective outcome measures of pain and function currently assessed in the patients with MPS. A single PRP treatment may alleviate muscle dysfunction caused by MPS. These preliminary results demonstrated the potential feasibility of using sEMG and MyotonPRO as tools for assessing the neuromuscular function of MPS.

## Introduction

Non-specific low back pain (LBP) is a common occurrence that affects individuals of all ages and is a major contributor to the global burden of disease^1^. Among the various causes of LBP, one of the most frequent is myofascial pain syndrome (MPS) which affects the spinal stabilizer muscles^2^. MPS has a high lifetime prevalence that affects approximately 30–85% of patients with musculoskeletal disorders^3^ and is usually found in the population aged from 27 to 50 years^4^. MPS is a chronic condition characterized by localized pain in the musculoskeletal system, often accompanied by palpable nodules and taut bands in the affected muscles, known as myofascial trigger points (MTrPs)^5^.

The MTrPs are hyperirritable spots, usually within a taut band of muscle fibers or in the muscle fascia which are painful on compression and can give rise to characteristic referred pain, motor dysfunction, and autonomic phenomena^6^. The development of MTrPs is primarily attributed to muscle tissue injury caused by factors such as trauma, abnormal posture, sports injuries, and dysfunction of the motor endplate^7^. Travell and Simons proposed the “energy crisis theory” to explain the formation of MTrPs^8^. Specifically, dysfunction of the motor endplate leads to an excessive release of acetylcholine^9^, which in turn causes sustained contraction of skeletal muscle fibers, the formation of contraction nodules, and tissue tension. Further, persistent muscle contraction results in local ischemia, hypoxia, and increased metabolic activity, triggering the release of a multitude of pain-inducing substances within the affected tissues and forming a vicious cycle^10^. MTrPs can be further categorized as active or latent. Active MTrPs are responsible for clinical symptoms, and their referred pain reproduces the patients’ reported symptoms. On the other hand, latent MTrPs may cause motor dysfunction such as stiffness, limited range of motion, and fatigability, but do not typically produce spontaneous sensory symptoms unless they are stimulated^11^. However, Quintner et al.^12^ claimed that the explanation for MPS caused by MTrPs lacks external validity.

Clinically, the identification of MTrPs and the assessment of muscle tone and tissue stiffness during a physical exam are commonly done by manual palpation, but this approach is subjective, requires experienced operators and different inter-rater reliabilities have been reported^13^. Recently, several objective techniques have been developed to quantitatively identify muscle stiffness by distinguishing the harder MTrPs from the surrounding normal muscle, including shear wave elastography^14^ and magnetic resonance elastography^15^. However, as these techniques are expensive and require technical expertise and complicated procedures, they are still not widely used in clinical practice and research. Despite the limited external validations, the diagnosis of MPS and its treatment protocols have been widely accepted by many practitioners. Therefore, it is necessary to have more convenient methods to objectively diagnose and quantify the physical and physiological characteristics of MTrPs based on two common manifestations of MPS (*i.e.*, motor endplate dysfunction and tissue tension)^8^, to clarify the pathophysiology of MTrPs and improve treatment.

The electrophysiology of MTrPs typically generates sustained, rapid, and low-amplitude abnormal electrical signals, such as spontaneous electrical activity or endplate noise, which can be detected using needle electromyography (EMG)^16^. The presence of MTrPs can be demonstrated by an increase in resting muscle activity^17^. Surface EMG (sEMG) is a non-invasive tool commonly used to evaluate neuromuscular function. Wytrążek et al.^13^ observed that MTrPs evoked an increase in sEMG amplitude at rest, but did not affect sEMG amplitude during maximal voluntary contraction (MVC). In contrast, according to Ge et al.^11^, during shoulder abduction at 25% MVC, the EMG activity in the upper trapezius muscle significantly increased when latent MTrPs were present compared to non-MTrPs. Currently, there is no consensus about the changes in sEMG activity in MTrPs. More recently, a new handheld, portable, non-invasive MyotonPRO device (Myoton AS, Tallinn, Estonia) has already proven to be objective and reliable in measuring biomechanical parameters of muscle properties^18,19^, and its validity has been widely confirmed^20,21^. The MyotonPRO employs a measurement method defined as the mechanical dynamic response method^19^. Studies have demonstrated that parameters of MyotonPRO, such as frequency, stiffness, and decrement, can be used to distinguish the MTrPs and non-MTrPs region of the infraspinatus in subjects with chronic shoulder pain^22,23^.

There is currently no standard treatment for patients with MPS. Clinically available treatments are trigger point injections, dry needling, manual therapy, physical exercise, and self-myofascial release^10,24,25^. Ultrasound-guided interventional procedures have recently been receiving increased attention as a therapy for treating MPS^26^. Platelet-rich plasma (PRP) is a concentrated platelet derived from whole blood after centrifugation, which can promote tissue repair and regeneration by utilizing the growth factors in autologous platelets and can regulate inflammation and immunity^27,28^. Studies have confirmed that PRP injection has a significant therapeutic effect on chronic tendon injury^29^ and chronic muscle injury^30^, but not on acute muscle injury^31^. Recently, Agarwal et al. demonstrated that PRP appears to be a more effective treatment approach compared to dry needling in the management of MTrPs in MPS patients^32^. Sakalys et al. also proved that PRP injections more effectively relieve pain in MPS of masseter muscle than lidocaine injections^33^. The application of PRP in the treatment of MPS presents evident feasibility and potential mechanisms, yet further research is required to determine its long-term effects and optimal application strategies.

Given the clinical significance of assessing neuromuscular function in MPS, the primary purpose of this study was to compare the differences in biomechanical parameters and muscle electrical activity between the painful and non-painful sides in patients with unilateral MPS. Additionally, this study aimed to evaluate the effects of PRP on muscle electrical activity and biomechanical parameters in patients with MPS, determine the feasibility of sEMG and MyotonPRO to assess the neuromuscular function of MPS and to evaluate the therapeutic effects of PRP.

## Materials and methods

### Study design

A non-randomized single-group design trial was implemented in the Department of Rehabilitation Medicine Center of West China Hospital Sichuan University from October 2022 to October 2023. The Institutional Review Board of the West China Hospital of Sichuan University authorized this work from the ethical point of view (WCHSCU_2023_99). The protocol was registered at the Chinese Clinical Trial Register (ChiCTR2300074199) as a clinical trial.

### Participants

Forty participants with unilateral lumbar MPS were recruited randomly. The following inclusion criteria were applied: (1) aged 18–70 years, (2) males or females, (3) meet the diagnostics of MPS: (1) a tender spot located with palpation, with or without referral of pain; (2) recognition of symptoms by the patient during palpation of the tender spot; and (3) at least three of the following: (a) muscle stiffness or spasm, (b) limited range of motion of an associated joint, (c) pain worsening with stress, and (d) palpation of a taut band and/or nodule associated with the tender spot. 4) Symptoms persist for more than six months. The exclusion criteria were: (1) any neurological signs consistent with nerve root compression; (2) a history of lumbar surgery or traumatic event; (3) pregnancy; or (4) known malignancy. After being advised of the purpose and potential risks of the study, all participants provided written informed consent and conducted in accordance with the Declaration of Helsinki.

### PRP preparation and injection

**Fig. 1:**
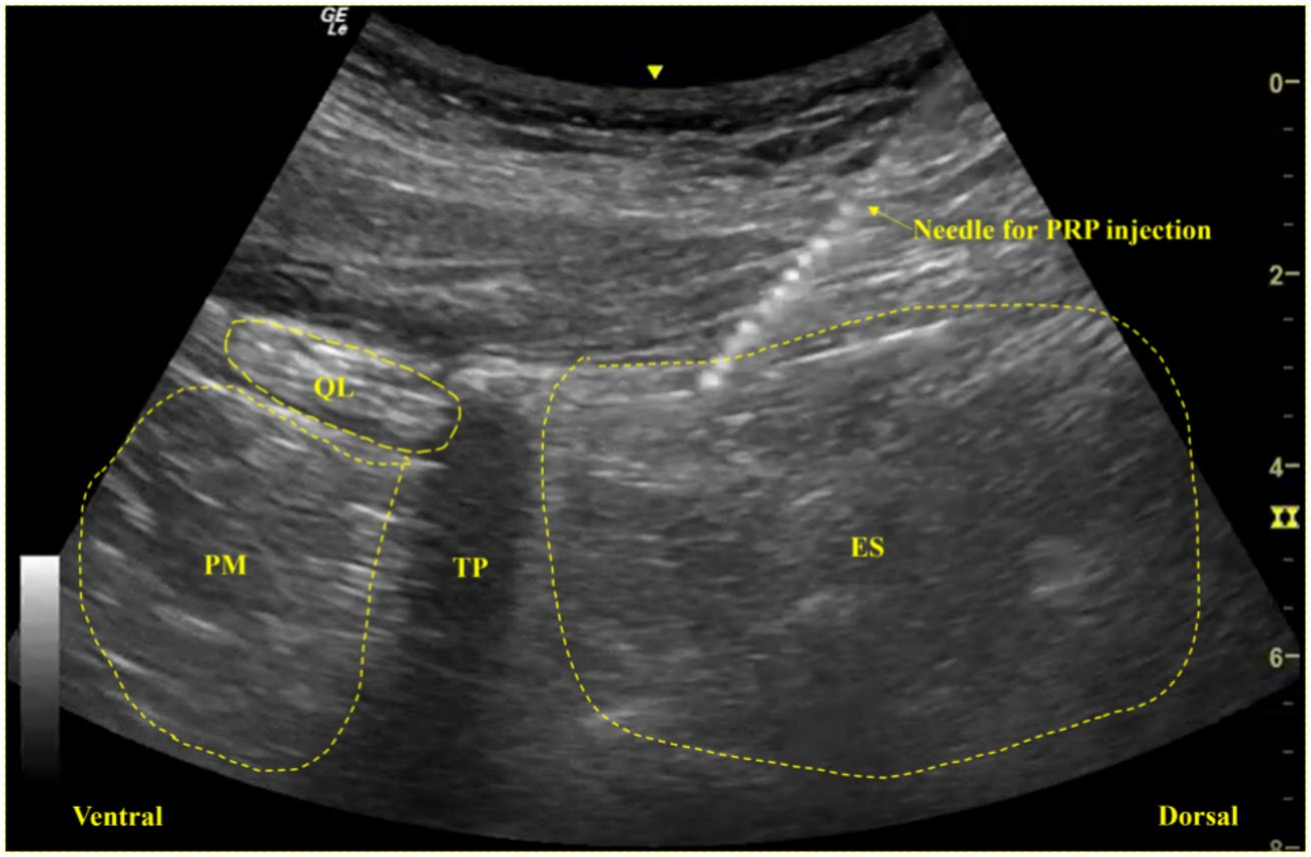
The schematic of the ultrasound-guided injection of platelet-rich plasma (PRP). PM: Proas major muscle; QL = Quardratus lumborum muscle; ES = Erector spinae muscle; TP = Transverse process.

Approximately 40 mL of whole blood was drawn from the elbow vein using a specialized 20G needle. The blood was then mixed with an anticoagulant (4 mL, 0.04 g/mL) at a ratio of 9:1 to prevent the formation of microbubbles and was distributed into an anticoagulant vacuum tube. PRP was prepared using commercially available kits (WEGO Platelet-Rich Plasma Preparation Kits, WEGO Ltd. Shandong, China). The centrifugation took place at room temperature. The sample was centrifuged at 1400 rpm for 10 min to obtain the buffy coat, followed by a second centrifugation at 2350 rpm for 5 min. After removing the supernatant, approximately 6 mL PRP would be obtained. Calcium chloride and thrombin were added to keep the bio-activity of PRP. The prepared PRP was not cooled before centrifugation and was immediately injected after preparation. Ultrasound-guided injection of PRP in lumbar erector spinae muscle (ES) targeted both MTrPs and their surrounding myofascial with meticulous layer-by-layer release (Fig. 1). The injection was performed by an experienced rehabilitation physician with more than 10 years of experience.

### Outcome measures

All outcome measures were assessed at baseline (before the treatment) and two weeks after the treatment.

#### Clinical assessment

The pain severity of the participants was evaluated using the visual analog scale (VAS), and the Oswestry Disability Index (ODI) was used to evaluate the severity of disability and disruption to lifestyle activities caused by LBP pain.

#### Biomechanical assessment

**Fig. 2:**
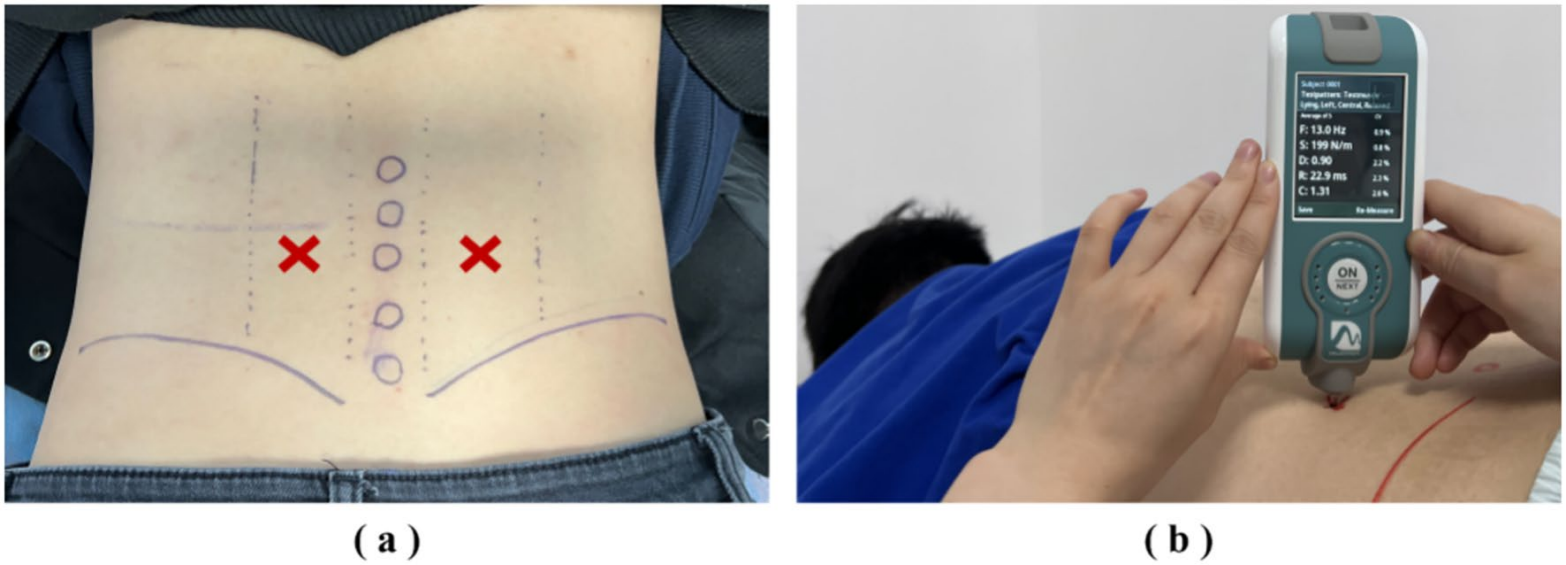
Biomechanical assessment. **a** Measurement position of MyotonPRO. **b** Measurement process of MyotonPRO.

Participants lay prone on the bed and were instructed to completely relax. The operator first marked the body surface by palpating the second lumbar vertebra (L2) muscle, the third lumbar vertebra (L3) muscle, and the fourth lumbar vertebra (L4) muscle, then the ES was marked with an oil pencil at 2.5 cm on both sides of the spinous process of L3 (see the points marked with a red cross in Fig. 2). Biomechanical parameters of the bilateral ES were recorded using a handheld, non-invasive MyotonPRO device (Myoton AS, Tallinn, Estonia). The following mechanical properties were measured: (1) *Oscillation frequency* [Hz], which characterizes the tone or tension of superficial skeletal muscles in their passive or resting state without voluntary contraction; The higher the value, the higher the muscle tone. (2) *Dynamic stiffness* [N/m] indicates the resistance of biological soft tissues to a force of deformation; The higher the value, the muscle becomes stiffer. (3) *Logarithmic decrement* is related to muscle elasticity; The lower the value, the muscle becomes more elastic. (4) *Relaxation time* [ms] characterizes tissue’s recovery time from displacement; The higher a tissue’s tension or stiffness, the faster a tissue recovers its shape, meaning the lower the value. (5) *Creep* is the ratio of relaxation and deformation time, which characterizes the gradual elongation of tissue over time when placed under constant tensile stress; The higher a tissue’s tension, structural integrity, or stiffness, the higher its resistance to creep, meaning the lower the value. To ensure accuracy, the MyotonPRO measurements were conducted by two experienced physiotherapists (N.L. and Q.W.). Five values measured by the same therapist at the same position were averaged as the final value.

#### Electromyography

**Fig. 3:**
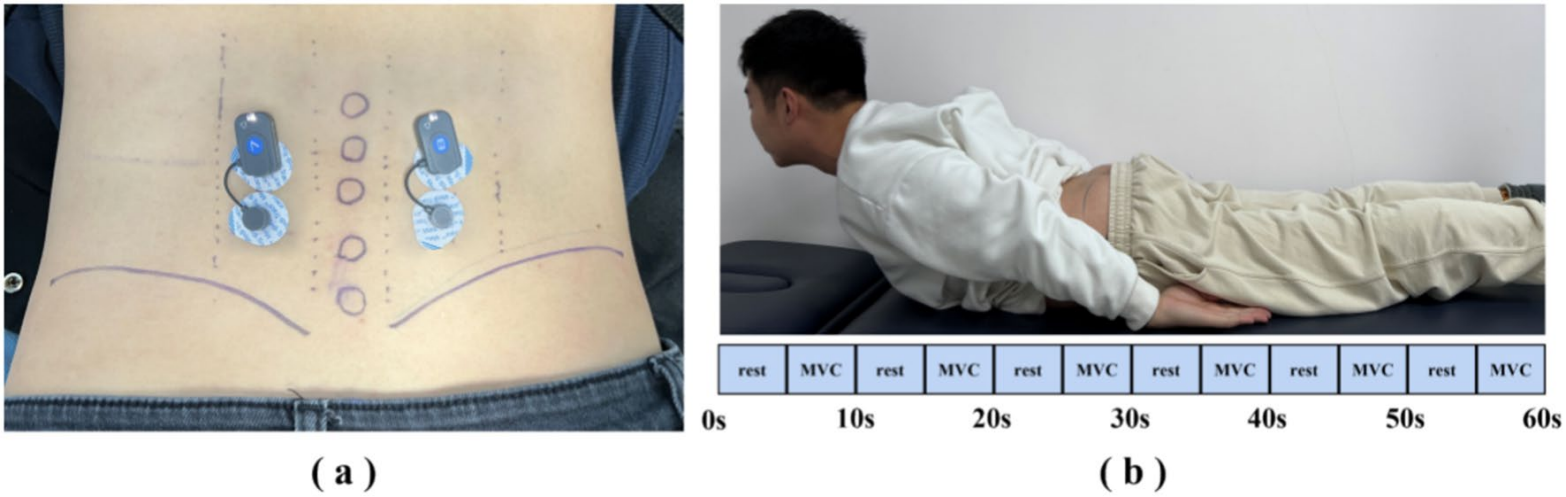
Experimental protocol. **a** The position of the electrodes. **b** Test task demonstrated by researcher.

A wireless sEMG system (FREEEMG BTS, Milano, Italy) was used to record the sEMG signals at a sampling rate of 1000 Hz. Before the experiment, the skin of the lumbar area was shaved lightly and wiped with alcohol pads to provide a low skin impedance condition for sEMG signal acquisition. Two Ag/AgCl electrode pairs (Kendall™ 930, Cardinal Health Inc., Dublin, Ohio, USA) were attached to the muscle abdomen of both ES (see Fig. 3a). The participants were asked to perform an isometric trunk extension exercise (see Fig. 3b). The participants were prone in their relaxed position before each trial started. Then, the participants conducted six trials. Each trial consisted of a 5-s MVC and a 5-s rest period between contractions under verbal motivation from the experimenter. Subsequent data analysis used only the stable 3-s EMG data from each 5-s contraction.

The raw sEMG signals were digitally filtered by a 50-Hz notch filter and bandpass 3rd order Butterworth filter between 20 and 450 Hz. Then, the sEMG signals were segmented into a series of 200 ms sliding windows with an increment of 50 ms. Four time-domain features of sEMG were extracted in each sliding window, namely, root mean square (RMS), mean absolute value (MAV), integrated EMG (iEMG), and waveform length (WL). Additionally, the frequency domain features used are the median frequency (MDF) and mean power frequency (MPF). The average of each feature was computed across all six trials.

### Statistical analysis

The descriptive statistics are reported in the mean and standard deviation (± SD) format. The Shapiro–Wilk test was used to assess the normality of the data distribution. Additionally, the assumption of homogeneity of variances was investigated using Levene’s test. To compare the differences in clinical assessment, sEMG features, and biomechanical parameters between the painful side and the non-painful sides, as well as pre- and post-PRP treatment, statistical analyses were performed using a paired *t*-test, with a significance level of 0.05. All statistical analyses were performed using SPSS version 25.0 (SPSS, Inc., IBM, Chicago, IL, United States).

## Results

### Participants characteristics

During the baseline data collection, a total of 40 participants with unilateral lumbar MPS were recruited and participated in the pre-treatment measurement, but only 33 participants with MPS on pailful side received PRP intervention and completed full procedures due to conflicting schedules. Thus, comparisons between the painful and non-painful sides were conducted with data from 40 participants (40/40), whereas pre- and post-PRP treatment comparisons were based on the 33 participants who completed full procedures (33/33). The characteristics of the participants are described in Table 1.

**Table 1:** Descriptive statistics of all participants represented as mean (± SD).

	Pre-treatment			Post-treatment		
	T (n = 40)	F (n = 23)	M (n = 17)	T (n = 33)	F (n = 20)	Male (n = 13)
Age (years)	42.2 ± 4.7	40.2 ± 4.7	43.6 ± 5.2	41.8 ± 4.5	42.2 ± 4.4	40.6 ± 4.8
Height (cm)	161.7 ± 4.5	154.7 ± 4.5	166.5 ± 6.3	162.1 ± 4.2	155.6 ± 4.3	166.1 ± 6.1
Weight (kg)	60.5 ± 6.5	59.5 ± 6.4	61.6 ± 11.9	61.1 ± 6.2	60.5 ± 6.6	61.9 ± 11.1
BMI (kg/m^2^)	23.4 ± 4.8	24.6 ± 5.5	22.5 ± 3.7	23.4 ± 4.8	25.1 ± 4.5	22.4 ± 4.6

T: total; F: female; M: male; BMI: body mass index; SD, standard deviation.

### Clinical assessment

**Fig. 4:**
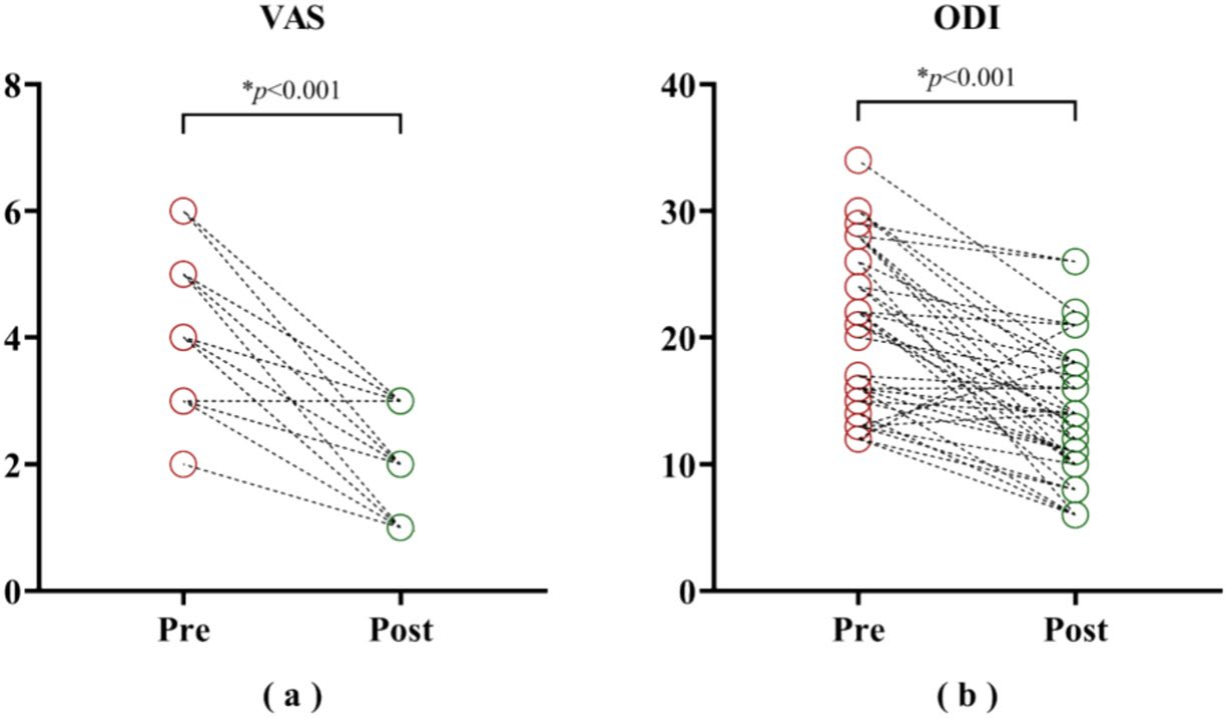
Clinical measurements pre- and post-intervention. Lower values of both VAS and ODI indicate lower severity of perceived pain. * indicates statistical significance at *p* < 0.05.

Statistically significant decreases were observed in both post-treatment VAS levels (pre: 4.3 ± 1.0; post: 2.2 ± 0.8; *p* < 0.001) and ODI levels (pre: 20.8 ± 6.5; post: 13.8 ± 5.4; *p* < 0.001) compared to pre-treatment (Fig. 4). Evidently, the clinical assessments indicated a significant reduction in MPS pain level following the PRP treatment.

### Biomechanical analysis

#### Baseline data

**Fig. 5:**
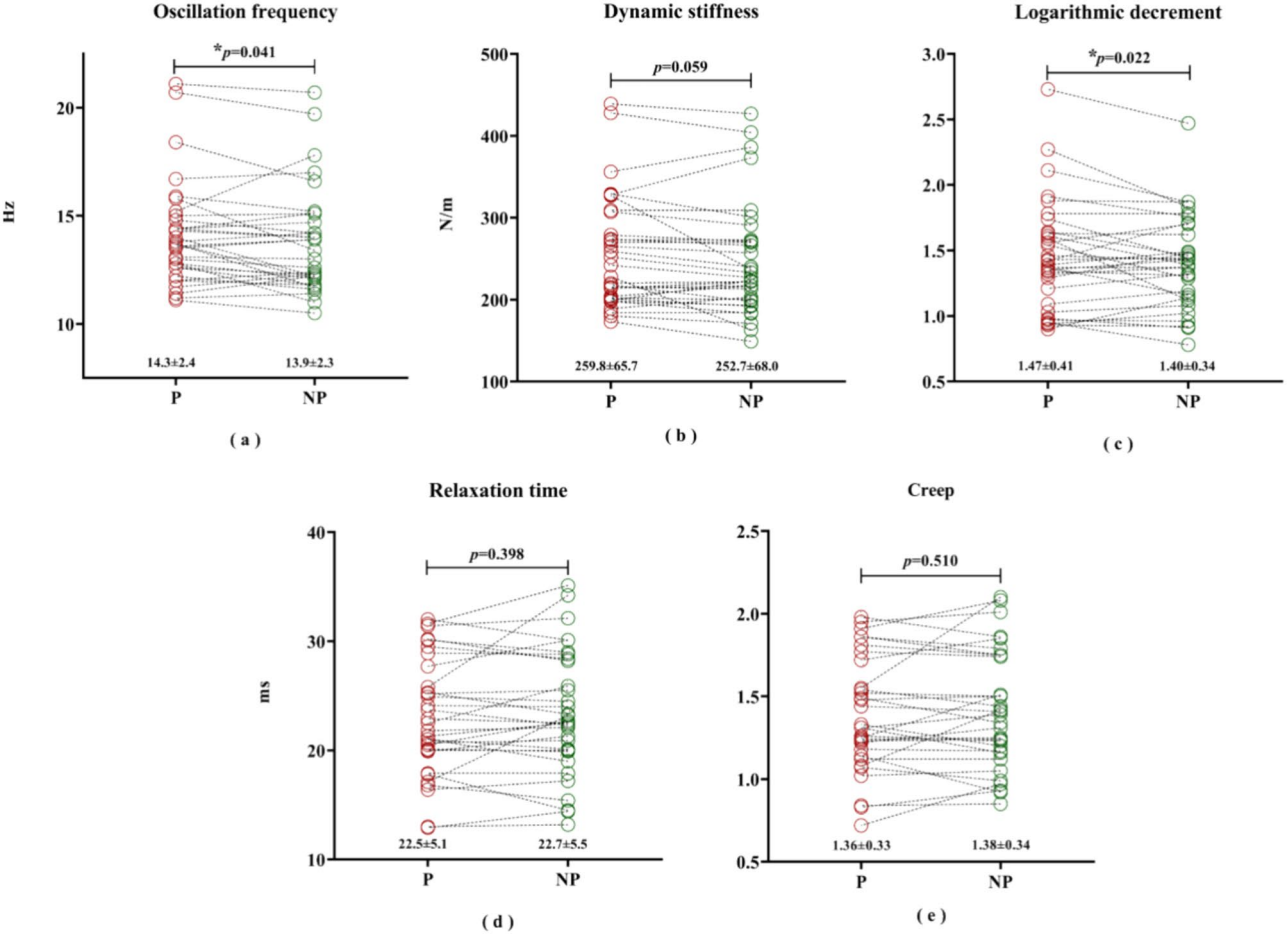
Biomechanical parameters of the painful side and non-painful side, before the intervention. * indicates statistical significance at *p* < 0.05. P: painful side; NP: non-painful side.

The paired *t*-test analysis showed that before PRP treatment, oscillation frequency reflecting muscle tension at rest (*p* = 0.041, see Fig. 5a) and logarithmic decrement (*p* = 0.022, see Fig. 5c) reflecting muscle elasticity were significantly higher than those on the non-painful side. This indicates that the painful side generally has higher muscle tension and lower muscle elasticity. However, there were no significant differences in relaxation time (*p* = 0.398, see Fig. 5d) and creep (*p* = 0.510, see Fig. 5e) which reflect the viscoelastic properties of muscles, and in dynamic stiffness (*p* = 0.059, see Fig. 5b) reflecting muscle stiffness.

#### Post-treatment

**Fig. 6:**
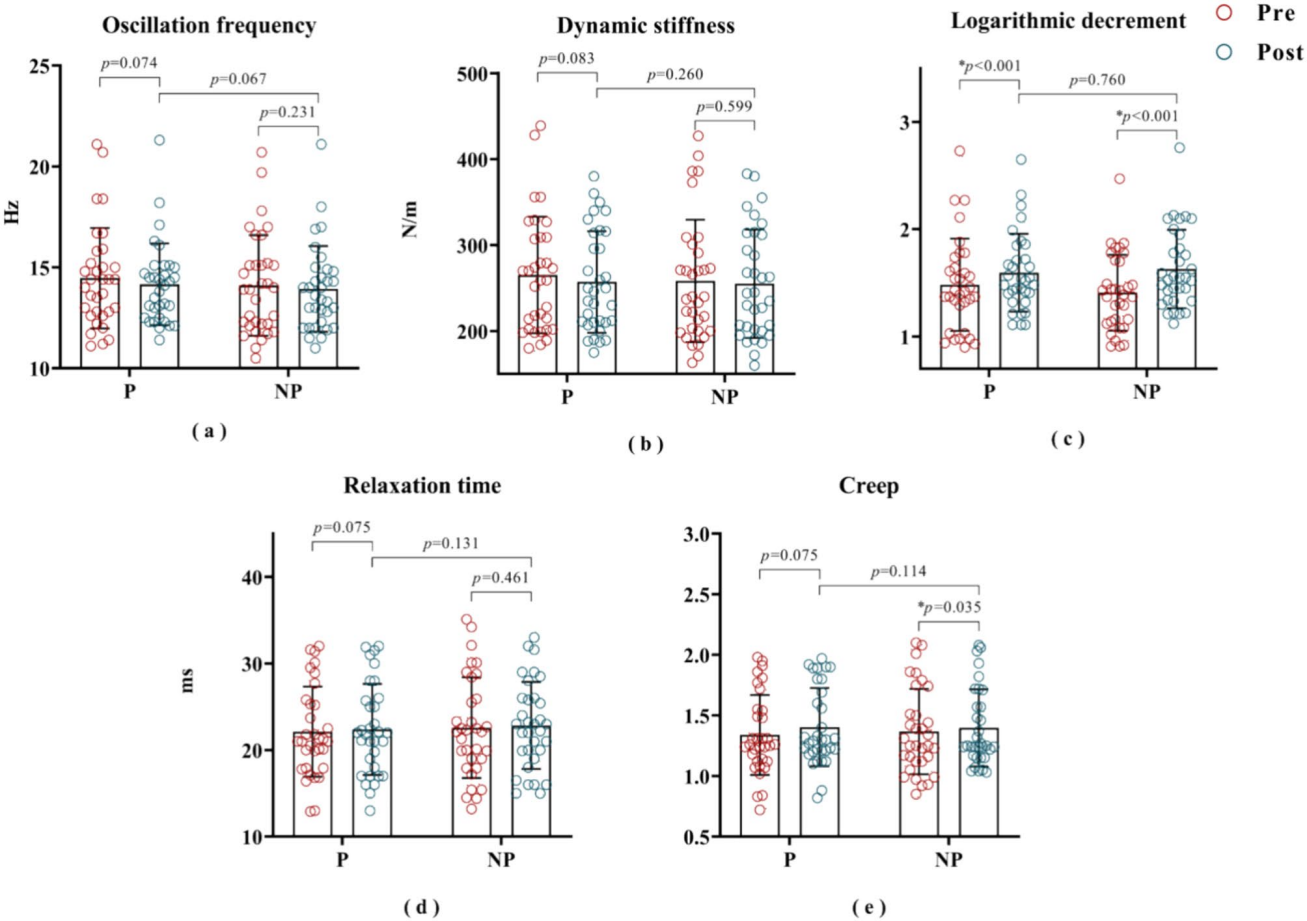
Biomechanical parameters of the painful side and non-painful side, before and after the intervention. * indicates statistical significance at *p* < 0.05. P: painful side; NP: non-painful side; Pre: pre-treatment; Post: post-treatment.

The logarithmic decrement on the painful side was significantly higher after treatment than before treatment (pre: 1.48 ± 0.43; post: 1.59 ± 0.36; *p* < 0.001), but there was no statistical difference in logarithmic decrement between the painful side and the non-painful side after treatment (painful side: 1.59 ± 0.36; non-painful side: 1.63 ± 0.37; *p* = 0.076), see Fig. 6c. Furthermore, the logarithmic decrement (pre: 1.40 ± 0.35; post: 1.63 ± 0.36; *p* < 0.001) and the creep (pre: 1.36 ± 0.35; post: 1.39 ± 0.32; *p* = 0.035) on the non-painful side was significantly higher after treatment than before treatment, see Fig. 6c,e. However, the remaining parameters were not statistically different between before and after treatment, or between the painful side and the non-painful side (*p* > 0.05), see Fig. 6a–e.

### Electrophysiological analysis

#### Baseline data

**Fig. 7:**
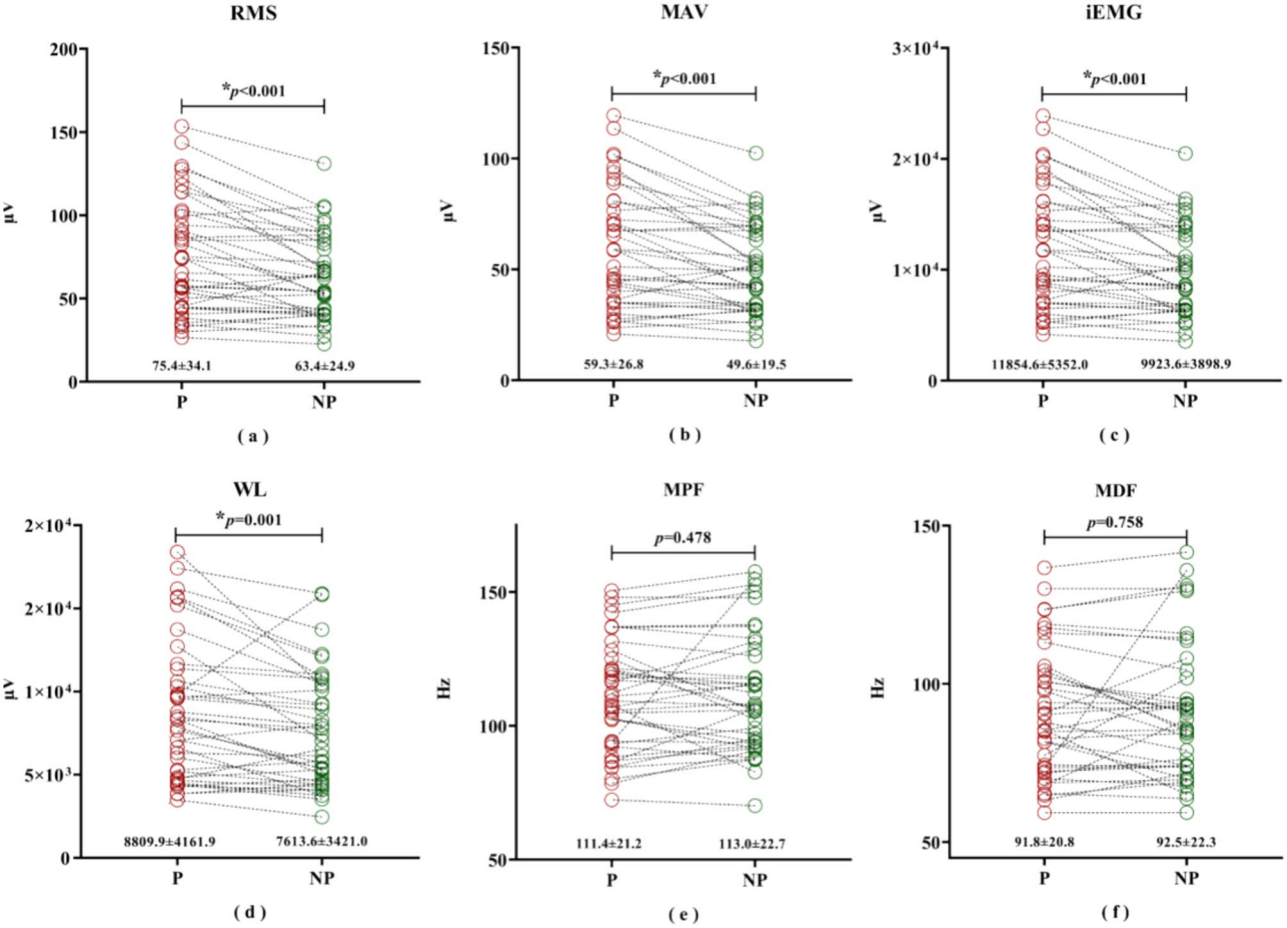
sEMG features of the painful side and non-painful side, before the intervention. * indicates statistical significance at *p* < 0.05. P: painful side; NP: non-painful side.

The time-domain features of the painful side were significantly higher than those in the non-painful side (RMS, *p* < 0.001; MAV,* p* < 0.001; iEMG,* p* < 0.001; WL,* p* = 0.001; see Fig. 7a–d). Additionally, there was no difference in frequency-domain features between the painful side and the non-painful side (MPF, *p* = 0.478; MDF, *p* = 0.758; see Fig. 7e,f).

#### Post-treatment

**Fig. 8:**
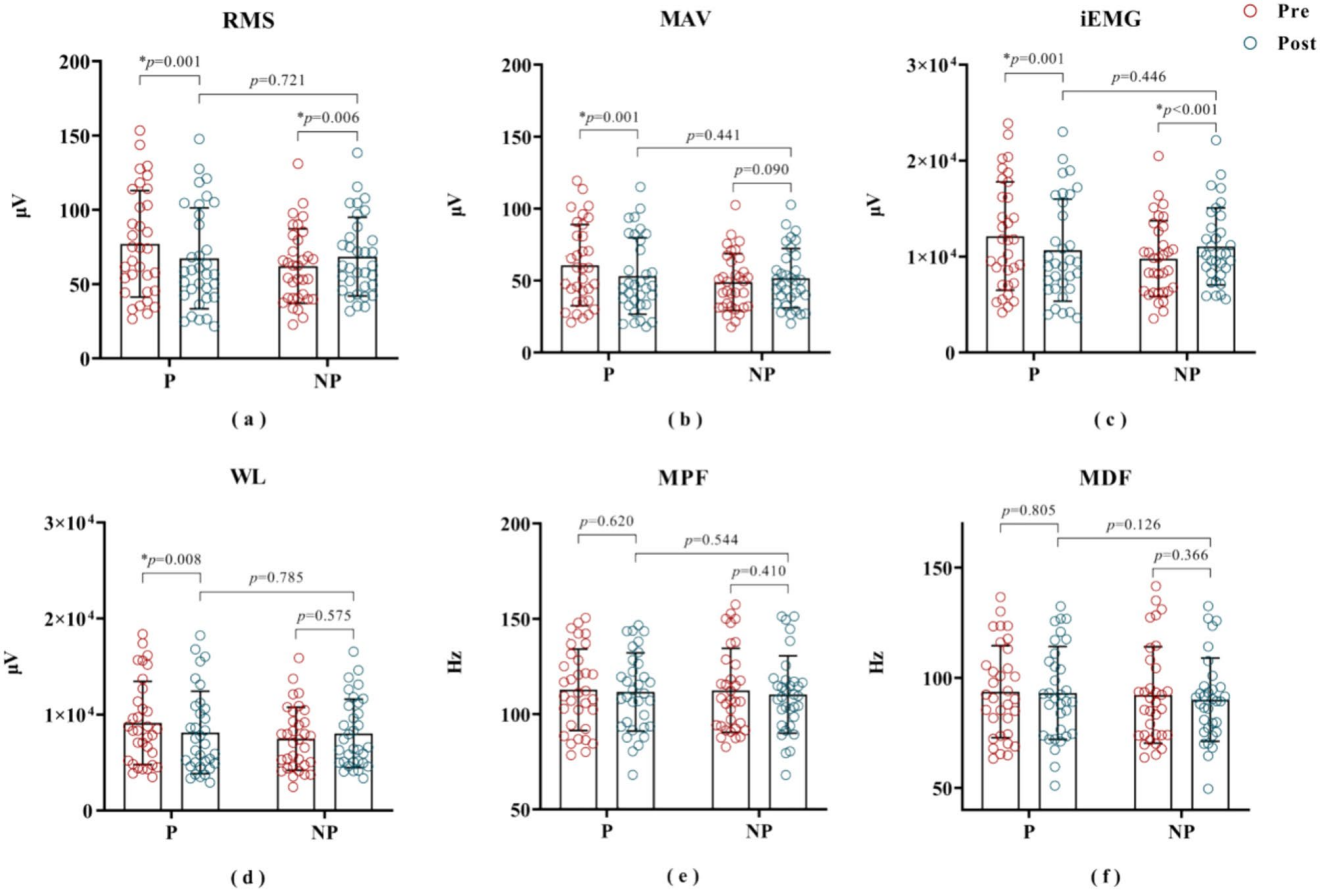
sEMG features of the painful side and non-painful side, before and after the intervention. * indicates statistical significance at *p* < 0.05. P: painful side; NP: non-painful side; Pre: pre-treatment; Post: post-treatment.

After PRP treatment, the features of the painful side reflecting sEMG energy, *i.e.*, RMS (pre: 77.1 ± 35.9; post: 67.3 ± 33.9; *p* = 0.001), MAV (pre: 60.7 ± 28.2; post: 53.1 ± 26.6; *p* = 0.001), iEMG (pre: 112131.2 ± 5642.8; post:10666.9 ± 5315.5; *p* = 0.001) and WL (pre: 9125.4 ± 4336.7; post: 8127.4 ± 4298.7; *p* = 0.008), decreased significantly, see Fig. 8a–d. In addition, the RMS (pre: 62.1 ± 25.1; post: 68.5 ± 26.5; *p* = 0.006) and iEMG (pre: 9769.5 ± 3955.7; post: 11049.7 ± 4006.6; *p* < 0.001) on non-painful side were significantly higher after treatment than before treatment, see Fig. 8a, c. However, there is no significant difference in the frequency-domain features of painful side before and after PRP treatment, see Fig. 8e, f. Obviously, there were no significant differences in all sEMG features between the painful side and non-painful sides after PRP treatment (*p* > 0.05), see Fig. 8.

## Discussion

Pain is a global public health problem that directly results in a decrease in functional activity^34^. The low back area is one of the most common sites for chronic pain^2^. Lumbar MPS is the most recurring type of LBP and a frequent cause for visiting a pain clinic^35^. MPS is seen most commonly between 27 and 50 years of age^4^. In our study, the mean age of the patients was 42 years old, which was consistent with other studies in the literature.

MTrPs can cause local motor dysfunctions such as muscle cramps and weakness, restricted joint range of motion, and altered motor control strategy^36^, and may lead to additional recruitment of motor units to compensate for a decreased discharge rate of low-threshold motor units, associated with muscle pain and muscle fatigue^37^. Manfredini et al. found that there was no significant difference in sEMG values of muscles on the painful and non-painful sides in patients with unilateral MPS of jaw muscles either at rest or during clenching tasks^38^. Ciubotariu et al.^39^ confirmed that during isometric contraction, muscle pain causes decreased force production, endurance time, and decreased activation of the painful muscle. Jiang et al.^40^ found that the RMS from the MPS group was slightly higher than that of the healthy group, but the MDF from the MPS group was lower than that of the healthy group. Our study, however, found that during MVC contraction, the muscle activity of the painful side was significantly higher than that of the non-painful side, while no difference in frequency-domain features between the two sides was found. There is no consensus on the effect of pain on the electrical activity of muscles. This may be due to differences in motor control strategy caused by pain, namely “tight control” and “loose control”^41^. Loose control protects against high muscle forces but eventually causes excessive tensile strains of tissues. On the contrary, tight control protects against large tissue strains from uncontrolled movement, but its long-term negative consequences can lead to sustained muscle activity. Based on the “motor control strategy” and “energy crisis theory”, the explanation for our findings may be that the changes in motor control induced by MPS may be more consistent with tight control, leading to sustained muscle activity resulting in abnormal increases in muscle electrical activity. However, this sustained muscle activity does not seem to lead to muscle fatigue.

Pain may cause the muscles on the painful side to remain in a contracted state for an extended period of time. This could be one of the reasons for increased muscle tone and stiffness^42^. Masaki et al. demonstrated that LBP-induced muscle spasms could cause an increase in multifidus muscle stiffness^43^. However, Kong et al. found that there were no significant differences in the stiffness of the back muscles between firefighters with and without LBP history^44^. Similarly, according to the “energy crisis theory”, the dysfunction of the motor endplates in MTrPs may contribute to the sustained contraction of skeletal muscle fibers and subsequent muscle cramping and tissue tension^8^. The results of this study found that the oscillation frequency and logarithmic decrement between the painful side and the non-painful side are different, which indicated that MPS may lead to higher muscle tone and lower muscle elasticity. This is consistent with previous research^45–47^.

Intramuscular injections are considered the recommended treatment for MTrPs^48^. Dry needling and local anesthesia are the most used modalities^49^. PRP is a novel treatment of MTrPs. As a method of delivering a high concentration of autologous growth factors and bioactive compounds at a low cost and with minimal invasiveness, it has become more popular^28^. In a study comparing the effectiveness of PRP injection versus dry needling for the management of MTrPs in the masseter muscle, the results indicated that PRP appears to be a superior treatment modality in managing MTrPs compared to dry needling ^32^. Another study compared the effectiveness of local anesthesia, botulinum toxin, and PRP injections for treating MTrPs in the masseter muscle, the results demonstrated that botulinum toxin injection seemed superior at the 3-month follow-up and remained effective up to 6 months^49^. The findings in the present study suggest that a single injection of PRP was enough to alleviate pain and improve the level of function in activities of daily living in patients with MPS. This is consistent with previous research^32,49^. It is noteworthy that our study revealed a notable reduction in muscle electrical activity on the painful side after PRP treatment. This could potentially be attributed to the disruption of existing contracture nodules and accelerated tissue repair caused by PRP injection targeting MTrPs. Additionally, the treatment may contribute to reduced inflammation, improved blood circulation, and inhibition of sustained muscle fiber contraction and continuous discharge^8^. There also has been some evidence that force can be transmitted through the muscular connective tissue and could lead to blockage of the spindle and/or its overexcitation^50^. This could also explain our findings of decreased sEMG activity after treatment. It is also noteworthy that our study found certain biomechanical parameters and sEMG features of the non-painful side changed, despite only the painful side receiving PRP treatment. This may be due to improved function on the painful side, resulting in reduced compensation on the non-painful side.

The present study has some limitations. Firstly, it did not include a control group, such as one that received no treatment or one that received only other treatment without PRP injection. Secondly, this study relied only on physicians’ experience in diagnosing MTrPs, lacking objective assessment methods, which likely resulted in heterogeneity due to the absence of MTrPs among the study population, and future studies should attempt to use ultrasound localization to reduce heterogeneity. Furthermore, relying only on verbal encouragement from the experimenter for participants to perform MVC contractions during trunk extension tasks may affect the consistency of sEMG results due to inconsistent muscle contraction states (dynamic or static). In the future, incorporating tasks such as resisted extension to control for the contraction condition could enhance the reliability of sEMG outcomes in both time domain and frequency domain. It is important to note that most of the current validity studies are related to the ‘dynamic stiffness’ parameter of the MyotonPRO devices. Therefore, conclusions based on the other parameters should be drawn with caution. Lastly, the present study only focused on the immediate effects of PRP, with no follow-up outcomes regarding PRP provided.

## Conclusion

This study made a preliminary investigation of the differences in muscle electrical activity and biomechanical properties between the painful and non-painful sides in patients with unilateral lumbar MPS and verified the feasibility of sEMG and MyotonPRO for assisting in MPS assessment. Our results indicated that MPS likely leads to abnormal time-domain features of sEMG rather than the frequency-domain features, and leads to increased muscle tone and decreased muscle elasticity. A single PRP treatment may alleviate muscle dysfunction caused by MPS. These preliminary results demonstrated the potential feasibility of using sEMG and MyotonPRO as tools for assessing the neuromuscular function of MPS.

## Data Availability

To request a copy of the data from this study, please contact the corresponding author.

## Abbreviations

LBP: Low back pain
MPS: Myofascial pain syndrome
MTrPs: Myofascial trigger points
sEMG: Surface electromyography
PRP: Platelet-rich plasma
VAS: Visual analog scale
ODI: Oswestry disability index
ES: Erector spinae muscle
RMS: Root mean square
MAV: Mean absolute value
iEMG: Integrated electromyography
WL: Waveform length
MDF: Median frequency
MPF: Mean power frequency
